# The Early Postnatal Nonhuman Primate Neocortex Contains Self-Renewing Multipotent Neural Progenitor Cells

**DOI:** 10.1371/journal.pone.0034383

**Published:** 2012-03-28

**Authors:** Jihane Homman-Ludiye, Tobias D. Merson, James A. Bourne

**Affiliations:** 1 Australian Regenerative Medicine Institute, Monash University, Clayton, Victoria, Australia; 2 Florey Neuroscience Institutes and Centre for Neuroscience, University of Melbourne, Melbourne, Victoria, Australia; Seattle Children's Research Institute, United States of America

## Abstract

The postnatal neocortex has traditionally been considered a non-neurogenic region, under non-pathological conditions. A few studies suggest, however, that a small subpopulation of neural cells born during postnatal life can differentiate into neurons that take up residence within the neocortex, implying that postnatal neurogenesis could occur in this region, albeit at a low level. Evidence to support this hypothesis remains controversial while the source of putative neural progenitors responsible for generating new neurons in the postnatal neocortex is unknown. Here we report the identification of self-renewing multipotent neural progenitor cells (NPCs) derived from the postnatal day 14 (PD14) marmoset monkey primary visual cortex (V1, striate cortex). While neuronal maturation within V1 is well advanced by PD14, we observed cells throughout this region that co-expressed Sox2 and Ki67, defining a population of resident proliferating progenitor cells. When cultured at low density in the presence of epidermal growth factor (EGF) and/or fibroblast growth factor 2 (FGF-2), dissociated V1 tissue gave rise to multipotent neurospheres that exhibited the ability to differentiate into neurons, oligodendrocytes and astrocytes. While the capacity to generate neurones and oligodendrocytes was not observed beyond the third passage, astrocyte-restricted neurospheres could be maintained for up to 6 passages. This study provides the first direct evidence for the existence of multipotent NPCs within the postnatal neocortex of the nonhuman primate. The potential contribution of neocortical NPCs to neural repair following injury raises exciting new possibilities for the field of regenerative medicine.

## Introduction

Under normal physiological conditions, the postnatal mammalian neocortex is widely accepted as a non-neurogenic zone. The prevailing dogma maintains that neurons residing within the postnatal neocortex are generated exclusively during embryogenesis [1]. With the exception of two recognized sites of postnatal neurogenesis, namely the subventricular zone (SVZ)/olfactory bulb axis and hippocampal dentate gyrus [2], the assertion that neurogenesis occurs in other regions of the postnatal brain, particularly the neocortex, remains highly controversial.

The principal methodology used to investigate evidence for postnatal neurogenesis has relied upon *in vivo* administration of thymidine analogues that label dividing cells. After a suitable ‘chase’ period, labelled cells that subsequently differentiate into postmitotic neurons have been identified on the basis of histological and/or neurochemical criteria, in particular, the co-expression of neuronal markers such as NeuN (reviewed by [3]). While numerous studies have found evidence to support low-level neurogenesis in the postnatal neocortex [4]–[7], many others have not [8]–[10]. The reasons for discrepancies between studies have been the subject of rigorous debate [1], [3], [11].

A complementary approach to investigate the intrinsic neurogenic capacity of the postnatal neocortex involves the *in vitro* characterization of acutely isolated neocortical cells to assay for the presence of multipotent neural progenitor cells (NPCs). The generation of proliferative clones of multipotent NPCs, known as neurospheres, by culturing acutely isolated cells in the presence of epidermal growth factor (EGF) and/or fibroblast growth factor-2 (FGF-2), provided critical evidence to establish the neurogenic potential of the adult mouse SVZ [12], [13] and hippocampus [14]. Assessing the neurosphere-forming capacity of the neocortex tissue has produced mixed results with early studies in the mouse suggesting the existence of FGF-2-responsive progenitors with latent neurogenic capacity [15] and more recent reports finding no such evidence in the normal uninjured neocortex [16].

In this study, we sought to determine the existence of neocortical NPCs within the primary visual cortex (V1, striate cortex) of the common marmoset monkey, a nonhuman primate that serves as a robust model for anatomical and physiological studies of the visual neocortex. Gestation in the marmoset monkey lasts 145 days and corticogenesis begins around embryonic day (ED) 50 similar to the macaque [17]. Cortical lamination starts after ED90, and by ED130 the six discrete cortical layers can be discriminated, albeit thinner than in the adult [18]. By birth, the pyramidal neuron maturation marker nonphosohorylated neurofilament (NNF) is expressed in all primary sensory areas, and is sequentially up-regulated in the rest of the neocortex as areas mature and refine their connections during the first postnatal months [19]. Onset of gliogenesis occurs just before birth and extends through the first postnatal weeks (reviewed in [20]).

We chose to examine evidence for neocortical NPCs in postnatal day (PD) 14 marmoset V1 because all neuronal cell layers within this region are generated well before birth [21] and pyramidal neurons exhibit adult-like characteristics defined by high level expression of the maturation marker NNF at this stage [22]. Furthermore, the visual cortex of the rat was among the earliest neocortical regions for which postnatal neurogenesis was described [7], and has been demonstrated to exhibit a robust neurogenic response to focal laser-lesioning [23]. Here we describe the identification of candidate NPCs in V1 neocortex expressing the progenitor marker Sox2 and proliferation marker Ki67 subpopulations of which express the Ng2 proteoglycan or GFAP and the *in vitro* characterization of V1-derived self-renewing neurospheres that exhibit multipotential differentiation capacity.

## Materials and Methods

### Ethics Statement

Experiments were conducted in accordance with the Australian Code of Practice for the Care and Use of Animals for Scientific Purposes and were approved by the Monash University Animal Ethics Committee (MAS-2009-08). Animals were obtained and housed at the National Nonhuman Primate Breeding and Research Facility (NNHPBRF; Monash University).

### Animals

Fifteen New World marmoset monkeys (*Callithrix jacchus*) aged postnatal day (PD) 14 were used in this study. Three animals designated for immunohistochemical analysis of the brain, were humanely killed with an overdose of pentobarbitone sodium (100 mg/kg; intraperitoneal injection) and the tissue was fixed by transcardial perfusion of PBS supplemented with heparin, followed by 4% paraformaldehyde.

For *in vitro* analyses, ablated V1 tissue was obtained from twelve animals that were used as part of a separate study.

### Isolation of primary visual cortex (V1) and neurosphere culture

V1 tissue was isolated from the left hemisphere of anesthetized PD14 marmoset monkeys (n = 12) by surgical excision in the coronal plane approximately at the level of Bregma -13.10 mm [24], as previously described [25]. Briefly, the animals were initially anesthetized with intramuscular injections (IM) of Alfaxan (12 mg/kg) and diazepam (3.0 mg/kg). After 15 minutes they were given antibiotic (procaine penicillin 25 mg/kg, IM) and the anesthesia was maintained with isoflurane (1–2% in 0.5 L/min oxygen). The plane of anesthesia was monitored throughout the procedure via pedal and corneal reflexes. The animal was placed on a thermostatically controlled heating pad to maintain core body temperature at 38°C, and blood oxygen level, heart rate, and temperature were monitored continuously throughout the experiment. After the procedure was completed, a topical local anesthetic gel (xylocaine 5% cream) was applied to the incision site to minimize discomfort in the period immediately after surgery. Post-operative analgesia (Temgesic 0.01 mg/kg; IM) was administered as required. As soon as the animal was alert and capable of supporting itself, it was returned to the family group and monitored on an hourly basis for over 8 hours. The animal was then monitored weekly for growth and normal development.

**Figure 1:**
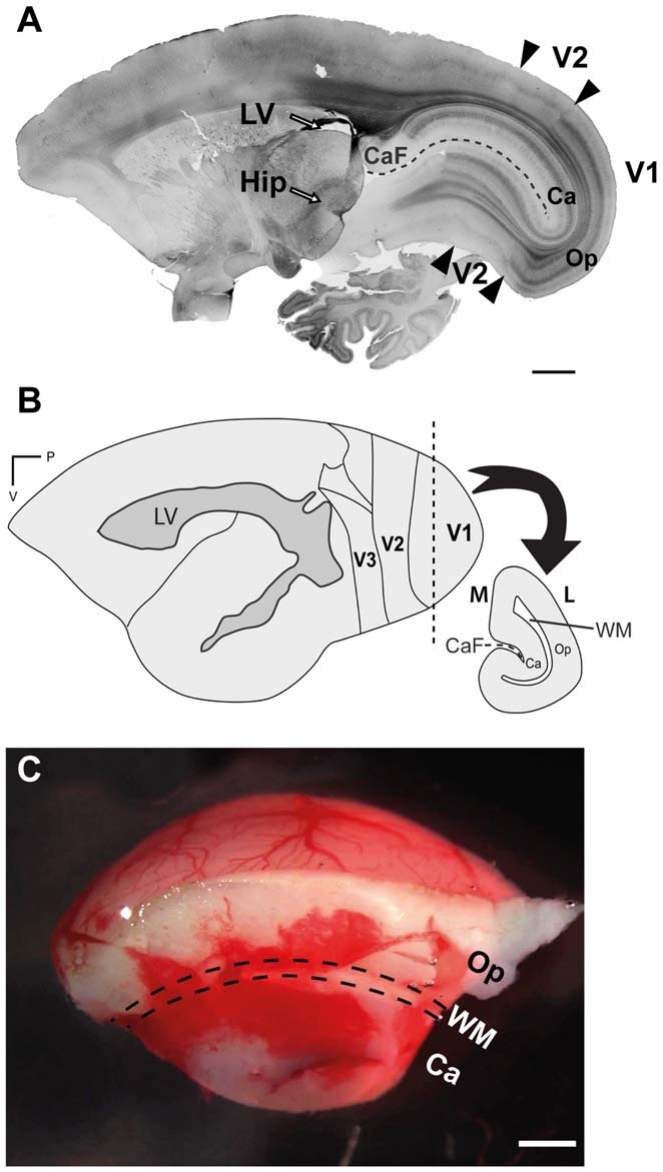
The neuronal maturation marker NNF is expressed by neurons in marmoset monkey V1 at PD14. ***A***, Parasagittal section reveals intense expression in layers 3 and 6 of V1, and the absence of labeling in adjacent area V2. ***B***, Schematic representation of the marmoset neocortex illustrating posterior visual areas. The vertical dashed line illustrates the plane of surgical excision used to isolate V1 tissue. The lateral ventricle is delineated by the gray shaded region based upon our own observations and the analyses of other groups [24], [39]. ***C***, Representative piece of resected V1 tissue subsequently processed for cell culture. Calcarine (Ca), calcarine fissure (CaF), hippocampus (Hip), lateral (L), lateral ventricle (LV), medial (M), operculum (Op), primary visual cortex (V1), secondary visual area (V2), white matter (WM). Scale bar = 2000 µm (***A***), 1000 µm (***C***).

The resected tissue constituted most of V1 including the outer operculum and the inner calcarine, measuring approximately 8×6×4 mm in size (**Fig. 1B, C**). The tissue was rinsed in ice-cold MEM (Gibco), the meninges were removed and the tissue was processed to a single cell suspension as described previously for mouse SVZ tissue [26]. In one experiment in which both the left and right V1 were excised from a PD14 marmoset, isolated V1 tissue was further microdissected to isolate operculum V1 by removing the underlying white matter and calcarine V1. Cells were resuspended in Neurocult NS-A proliferation medium (StemCell Technologies), viability was assessed by trypan blue exclusion and cells were seeded at 500cells/well into low attachment 96 well plates (Corning) in proliferation medium alone or with recombinant human FGF-2 (10 ng/mL; BD Biosciences) and/or recombinant human EGF (20 ng/mL; Peprotech). Growth factors were supplemented every 4 days.

#### Passaging

Every 28 days, neurospheres were harvested by centrifugation and a single cell suspension was prepared as previously described [26]. Cells were resuspended in 1 mL proliferation medium containing the appropriate growth factors and seeded at a density of 4.8×10^4^ cells/dish in 60 mm ultralow attachment culture dishes (Corning). Growth factors were supplemented every 4 days.

#### Differentiation assay

Single cell suspensions of dissociated neurospheres were plated onto poly-ornithine/laminin coated coverslips in a 24 well plate, as described previously [26]. Fifty microliters of each 1 mL cell suspension was seeded into the centre of each coverslip and incubated for 30 minutes to allow for cell attachment before addition of 500 µL of Neurocult NS-A differentiation medium (StemCell Technologies) containing 1% fetal calf serum (Invitrogen). Half of the medium was replaced every 4 days and the cultures were fixed with 4% PFA after 15 days *in vitro* (DIV).

#### Bromodeoxyuridine (BrdU) pulse

BrdU (0.2 µM) was added to the culture medium for 8 hours then cells were fixed in 4% PFA for 30 minutes at room temperature (RT). Neurospheres were rinsed, resuspended in OCT (TissueTek) and small blocks frozen down at −20°C. Sections of OCT-embedded neurospheres (12 µm thick) were collected on Superfrost Plus slides.

### Immunolabelling

Samples were blocked with 4% normal goat serum (NGS)/0.3% Triton X-100 in PBS for 30 minutes. Triton X-100 was omitted for O4 staining. For neurospheres (slide-mounted or grown on coverslips), primary antibodies were applied for 2 hours at RT. For free-floating cryosections of fixed marmoset brains (40 µm thick, parasagittal plane), primary antibodies were incubated at 4°C overnight. For BrdU immunolabelling, sections were incubated with 2 M HCl at 37°C for 15 minutes, washed in 0.1 M borate buffer (pH 8.5) and incubated with rat anti-BrdU antibody for 2 hours at RT. Primary antibodies were visualized using AlexaFluor-488 or 594-conjugated secondary antibodies (Invitrogen) incubated for 30 minutes in blocking solution. Nuclei were counterstained with Hoechst 33328 (Molecular Probes) then samples were slide-mounted and coversliped with mounting medium (Dako). NNF immunohistochemistry was performed as previously described [19].

Primary antibodies were as follows: mouse anti-MAP2 (1∶5000, Sigma), mouse anti-human nestin (1∶200, Millipore), mouse anti-nonphosphorylated neurofilament (1∶1,000, Covance), mouse anti-O4 (1∶25, DSHB), mouse anti-Sox2 (1∶25, R&D Systems), mouse anti-PCNA (1∶1000, Abcam), rabbit anti-Ki67 (1∶500, Dako), mouse and rabbit anti-GFAP (1∶1000, Millipore), rabbit anti-NG2 (1∶200, Millipore), rabbit anti-Tbr2 (1∶500, Millipore), goat anti-doublecortin (DCX, 1∶100, Santa Cruz), rat anti-BrdU (1∶20, AbD Serotec).

### Microscopy, image analysis and statistics

Samples were examined using a Zeiss Imager Z1 microscope fitted with the Apotome system allowing 3D analysis of sections. Images were captured using an Axiocam HRm digital camera with Axiovision 4.7.1 software (Zeiss). AdobePhotoshop CS3 10.0.1 was used for image adjustment and statistical analysis was performed using GraphPad Prism 5.0b for Macintosh.

## Results

We investigated the existence of multipotent NPCs residing within V1 of the marmoset monkey at PD14. We have previously demonstrated that neuronal maturation within V1 precedes that of other visuocortical areas at early postnatal ages [19]. We confirmed that at PD14, the maturation marker NNF was strongly expressed in layers 3 and 6 of V1 and was absent from adjacent V2 (**Fig. 1A**). Figure 1A also serves to illustrate the significant distance (>6.5 mm) separating V1 from the hippocampus and lateral ventricles, the two known neurogenic niches in the postnatal brain. The schematic (**Fig. 1B**) illustrates the anatomical level equivalent to Bregma -13.10 mm in the adult brain [24] at which surgical resection of V1 tissue was performed (**Fig. 1B**, vertical dashed line). Notably the most caudal limit of the lateral ventricle (**Fig. 1B**, gray shaded region) is observed at approximately Bregma -6.26 mm in the adult, thus excised V1 tissue contains no lateral ventricle that could potentially harbour resident NPCs. An example of resected V1 tissue is illustrated in **Figure 1C**.

### Proliferative neural progenitors are present in V1 neocortex at PD14

**Figure 2:**
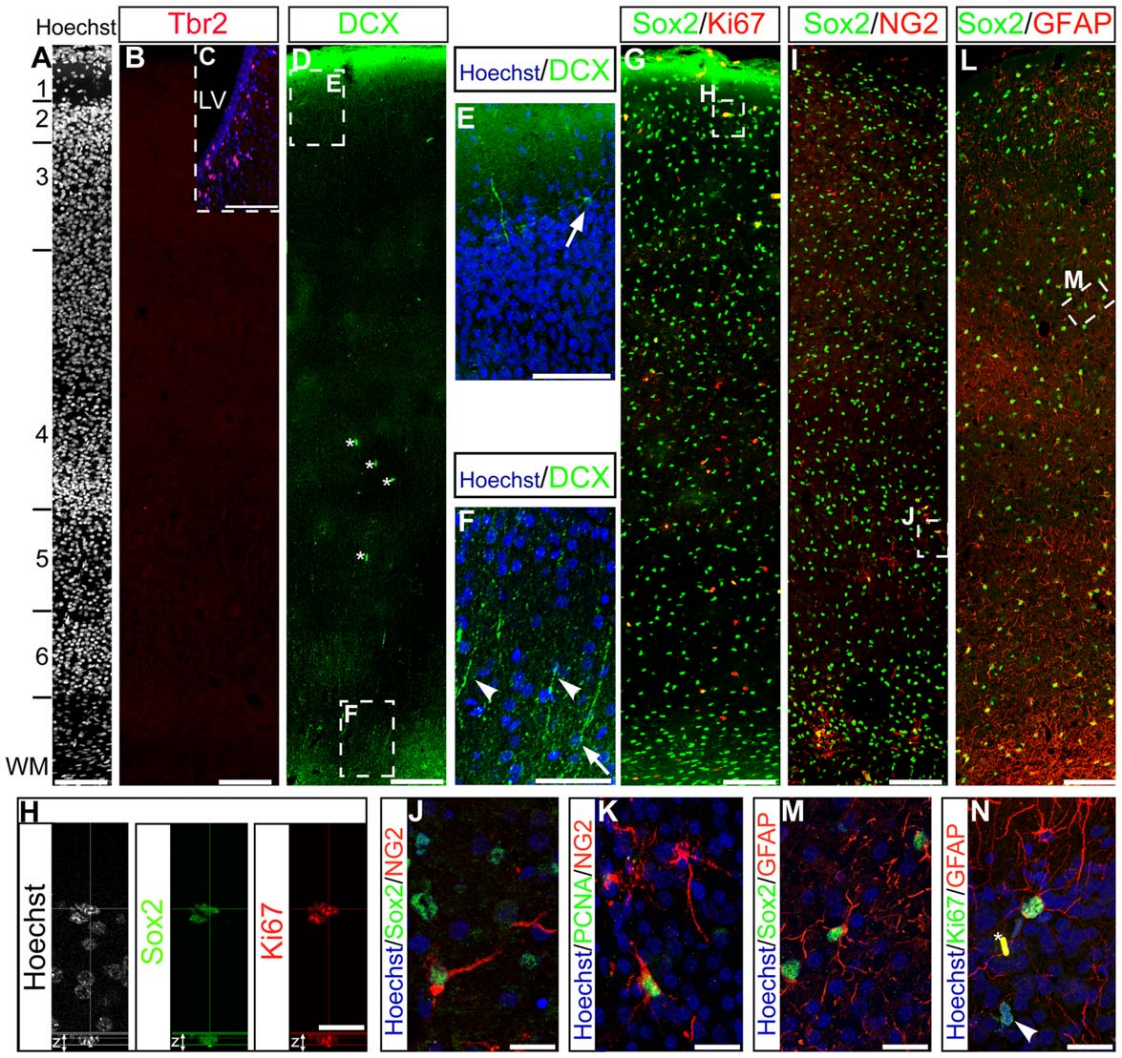
DCX, Sox2, Ki67, NG2 and GFAP are expressed in V1 at PD14. Hoechst staining demarcates cortical layers (***A***). Tbr2 was not expressed in V1 but expressed in the SVZ (***C***, LV:lateral ventricle). DCX^+^ cell bodies (***D***, *blood vessels) were located at the limit between layers 1 and 2 (***E***, arrow), and in white matter (***F***, arrow). DCX^+^ cells in the white matter extended long radial processes towards the outer layers (***F***; arrowheads). Sox2^+^ cells (***G***) were distributed across all cortical layers and white matter, of which a subset co-expressed Ki67 (***H***, *z* = z stack). NG2^+^ cells distributed in the white matter and the cortical layers co-expressed Sox2 (***I***, ***J***) and the proliferation marker PCNA (***K***). The majority of Sox2+ cells co-expressed GFAP (***L***, ***M***), a subset of astrocytes expressed Ki67 (***N***, *blood cell) however most of the Ki67^+^ cells were GFAP^−^ (***L***, arrowhead). Scale bar = 100 µm (***A, B, C, D, G, I, L***), 50 µm (***E***, ***F***), 20 µm (***H, J, K, M, N***).

To identify candidate NPCs in PD14 V1, we examined the expression of Tbr2, doublecortin (DCX), Sox2, NG2 and Ki67 in this region. The expression of the transcription factor Tbr2, highly expressed in the SVZ (**Fig. 2C**) and the hippocampus, was not detected in V1 (**Fig. 2B**), suggesting the absence of SVZ-derived intermediate progenitors in the V1 at PD14. Two distinct populations of DCX^+^ cells were identified (**Fig. 2D**). Multipolar DCX^+^ cells were located at the interface between layers 1 and 2 that extended processes into layers 1 and 3 (**Fig. 2E**, arrow). A separate population of DCX^+^ cells with radial morphology were located in the white matter that extended long processes into layer 6 (**Fig. 2F**, arrowheads). Since no DCX^+^ cells co-expressed the proliferation marker Ki67, we conclude that all DCX^+^ cells in V1 are postmitotic at PD14. Cells expressing Sox2, a transcription factor previously demonstrated to be required for the maintenance of NSCs in the rodent [27], were distributed homogenously throughout all cortical layers including the white matter, except for acellular layer 1 (**Fig. 2G**). A subpopulation of Sox2^+^ cells co-expressed Ki67^+^, defining a candidate population of proliferative progenitors *in vivo*, however not all Ki67^+^ cells were Sox2^+^ (**Fig. 2H**). Sox2^+^ cells were predominantly defined by co-expression of either NG2 proteoglycan or GFAP. Sox2^+^/NG2^+^ clusters of oligodendrocyte progenitor cells were distributed in the white matter and the cortical layers (**Fig. 2I, J**). A subset of cells expressing NG2 were proliferative, as revealed by co-expression of PCNA (**Fig. 2K**). The astrocytic marker GFAP was extensively expressed across all the cortical layers (**Fig. 2L**) and highly co-localised with Sox2 (**Fig. 2M**). A small fraction of the GFAP^+^ astrocytes remained mitotic at that stage as revealed by co-expression of Ki67, but not all Ki67^+^ cells expressed GFAP (**Fig. 2N**).

### V1 contains multipotent EGF and FGF-2-responsive neurosphere-forming cells

**Figure 3:**
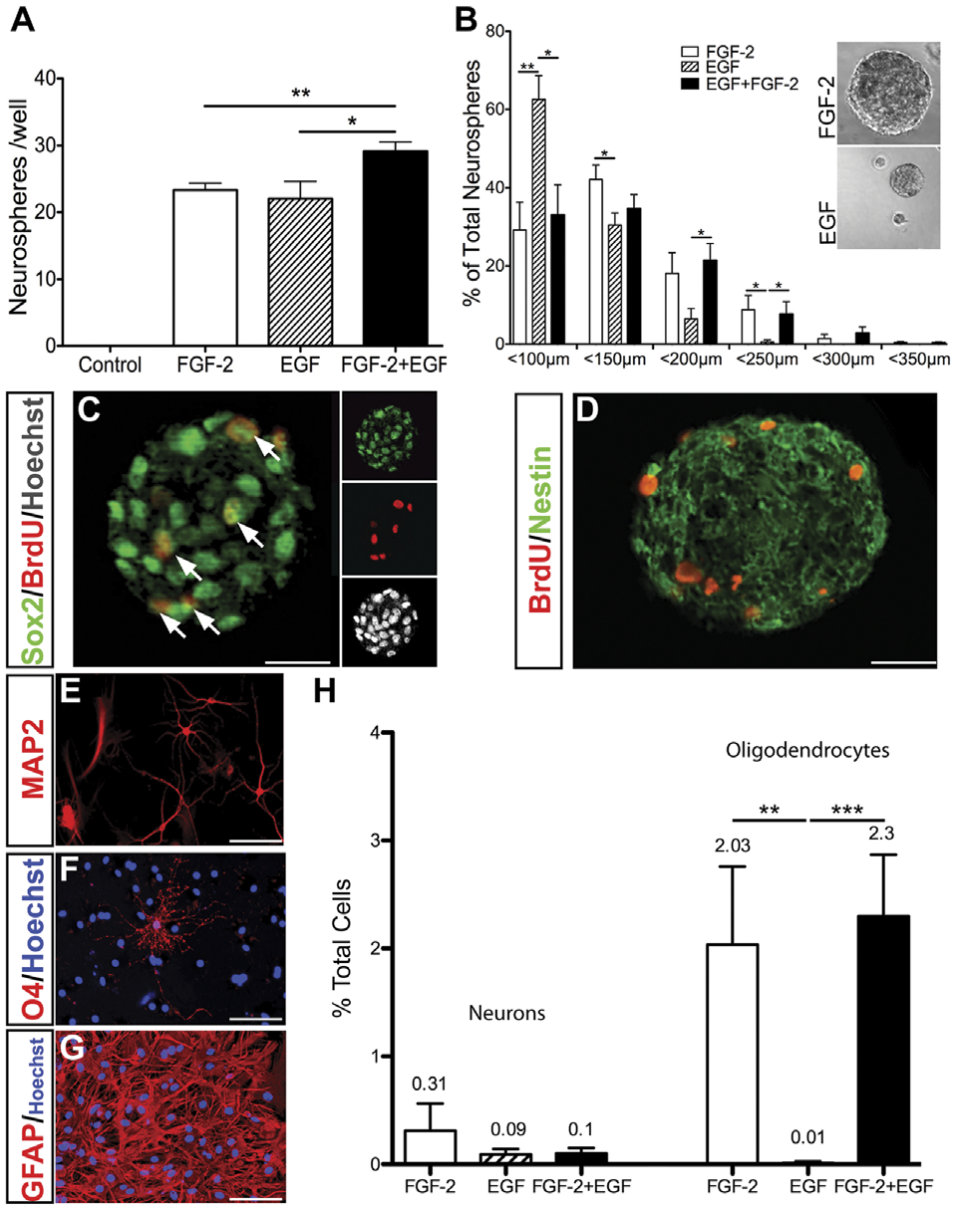
EGF and/or FGF-2 stimulate the formation of multipotent neurospheres from dissociated V1 tissue. Plot of primary neurospheres counted in each well of a 96 well plate after 15 DIV. Acutely isolated cells seeded at 500 cells/well (n = 11 independent isolates per condition) (***A***). Plot of mean primary neurosphere diameter (n = 6 independent isolates per condition) (***B***). After 15 DIV, neurospheres were pulsed with BrdU for 8 hours prior to fixation. BrdU^+^ cells found within the neurospheres (***C***, ***D***, red) were double-labelled with Sox2 (***C***, arrowheads), and expressed Nestin (***D***). Differentiation of dissociated primary neurospheres for 15 DIV generated MAP2^+^ neurons (***E***), O4^+^ oligodendrocytes (***F***) and GFAP^+^ astrocytes (***G***). Percentages of MAP2^+^ neurons and O4^+^ oligodendrocytes compared to total differentiated cells (***H***). Data represent mean ± SEM (*** *P*<0.0001; **P<0.009, *P<0.03; Mann-Wittney *U* test ***A,B***; Kruskal-Wallis test, ***H***).

To assess whether postnatal V1 contains NPCs capable of generating neurospheres following growth factor stimulation, acutely isolated V1 cells were cultured in proliferation medium alone or in the presence of FGF-2 and/or EGF (*n* = 11 independent tissue isolates per condition) (**Fig. 3A**). Whereas no neurospheres formed without growth factors, exposure to FGF-2 or EGF induced the formation of 23.34±0.16 and 22.3±0.3 neurospheres/well, respectively. Compared to FGF-2 or EGF alone, co-stimulation with FGF-2+EGF significantly increased neurosphere generation to 29.15±0.2 neurospheres/well (P<0.009 and P<0.03, respectively, Mann-Whitney *U* test).

Although the frequency of neurosphere formation was equivalent for both EGF and FGF-2 alone, EGF-generated neurospheres were markedly smaller than FGF-2 or FGF-2+EGF-generated neurospheres (*n* = 6 independent isolates per condition) (**Fig. 3B**). Whereas 10% of FGF-2 or FGF-2+EGF-generated neurospheres were greater than 200 µm in diameter, fewer than 1% of EGF-generated neurospheres reached this size indicating reduced NPC proliferation with EGF alone.

To establish whether neurosphere-forming cells are localized specifically within operculum V1, in an additional experiment excised V1 tissue was further microdissected following surgical excision to remove the underlying white matter and calcarine V1 (n = 2 independent isolates). Single cell suspensions were assayed using the neurosphere assay in the presence of FGF-2 and EGF. Clonogenic frequency for isolated operculum V1 neocortex was 24.11±2.1 neurospheres/well closely reflecting the results obtained for V1 tissue comprising operculum and calcarine V1 and juxtaposing white matter. We also noted that the size of the neurospheres was comparable to FGF-2/EGF-generated neurospheres derived from non-microdissected V1. These results demonstrate that neurosphere-forming NPCs reside within the 6 cortical layers of operculm V1 and are not restricted to the juxtaposing white matter and/or calcarine V1.

To characterize the identity of cells within FGF-2+EGF-generated neurospheres derived from excised V1 tissue, cultures were pulsed with BrdU for 8 hours prior to neurosphere fixation and sectioning. Immunocytochemistry revealed that all Hoescht-labelled nuclei within neurospheres were Sox2^+^ and a subpopulation were also BrdU^+^ indicating the presence of mitotic NPCs (**Fig. 3C**, arrows). Nestin was also strongly expressed in a filamentous pattern throughout BrdU-incorporating neurospheres (**Fig. 3D**) enabling us to conclude that FGF-2+EGF-generated neurospheres comprised of predominantly undifferentiated NPCs.

To assess the intrinsic differentiation potential of V1 NPCs, we cultured dissociated primary neurospheres under differentiating conditions for 15 DIV. Immunocytochemical analysis revealed the presence of MAP2^+^ neurons (**Fig. 3E**), O4^+^ oligodendrocytes (**Fig. 3F**) and large numbers of GFAP^+^ astrocytes (**Fig. 3H**), thereby confirming the multipotentiality of NPC cultures (n = 3 independent cultures per condition). Commitment to an oligodendrocyte fate was growth factor-dependent with 2.03±0.73% of FGF-2-generated, 2.3±0.57% of FGF-2+EGF-generated and just 0.01% of EGF-generated neurosphere cells giving rise to O4^+^ oligodendrocytes. The generation of neurons was not growth factor dependent with 0.31±0.2% of FGF-2-generated, 0.1±0.05% of EGF-generated and 0.1±0.01% of FGF-2+EGF-generated neurosphere cells differentiating into MAP2^+^ neurons.

### V1 NPCs are self-renewing, clonal and multipotent over several passages

**Figure 4:**
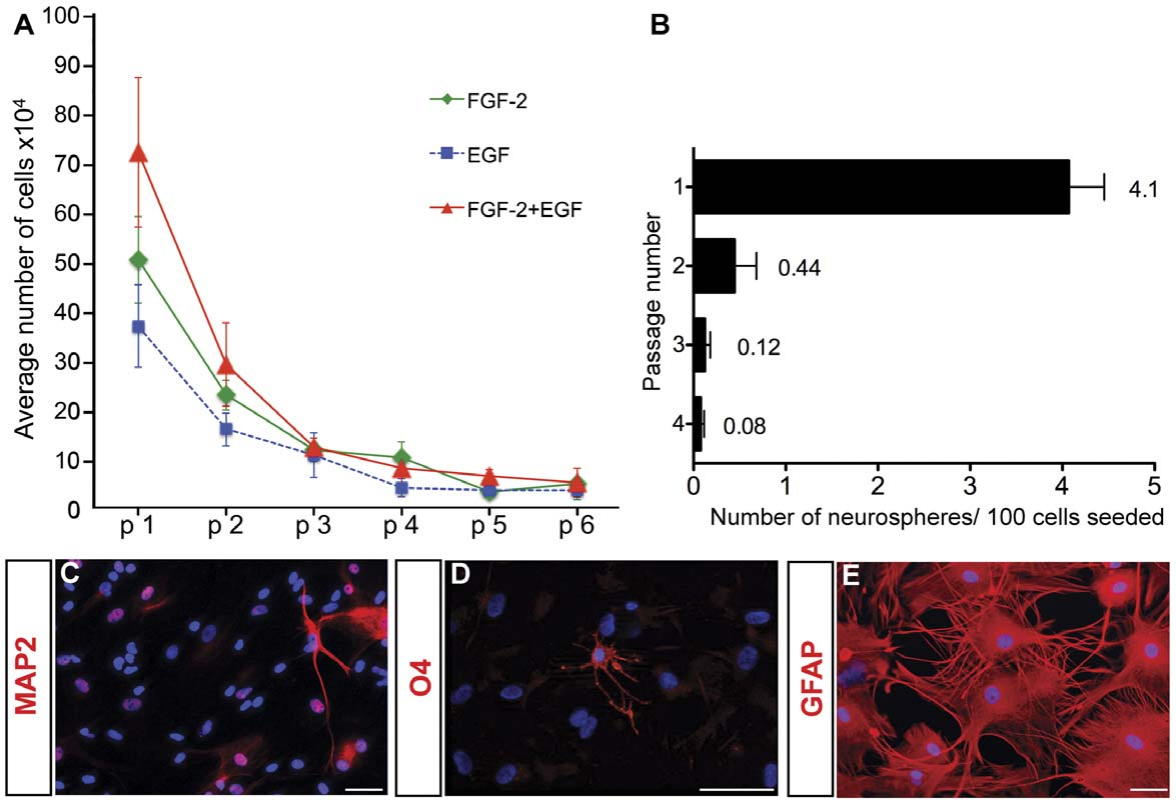
V1 derived neurospheres were maintained for at least 6 passages and remained multipotent from passage 0 to 3. Plot of cell yield at each passage based on a seeding density of 4.8×10^4^ cells per dish (n = 10 independent isolates per condition) (***A***). Clonogenicity assays of FGF-2+EGF-generated neurospheres at passages 1–4 (1,000 cell/cm^2^ seeding density) (***B***). At passage 3, neurospheres gave rise to MAP2^+^ neurons (***C***), O4^+^ oligodendrocytes (***D***) and GFAP^+^ astrocytes (***E***) after 15 DIV. Data represent mean ± SEM. Scale bar = 50 µm.

Next we assessed the self-renewal and proliferative properties of V1 NPCs to establish whether they exhibited properties of a long-term self-renewing NPC population. Primary neurospheres were dissociated into single cells and seeded at equal density (4.8×10^4^ cells per 60 mm dish) in the same growth factor conditions used for their initial derivation. Although secondary neurospheres were observed within 7 DIV for all growth factor conditions, slow growth delayed neurosphere passaging until 28 DIV. Neurospheres were passaged serially in this manner every 28 days for up to 6 passages. To assess the extent of cell proliferation at each passage, we plotted the number of cells harvested from each dish over time (**Fig. 4A**). At passage 1, we obtained 50.13±8.8×10^4^ cells (in FGF-2), 36.7±8.4×10^4^ (in EGF) and 71.8±15.1×10^4^ (in FGF-2+EGF) cells, representing an increase in total cell population of 10.4-fold in FGF-2, 7.6-fold in EGF and 15-fold in FGF-2+EGF. These data reflected both the increased clonogenic frequency of V1 cells cultured with FGF-2+EGF and reduced size of neurospheres cultured in EGF alone (**Fig. 3A,B**). By passage 2, population expansion was reduced to 4.7-fold in FGF-2, 3.3-fold in EGF and 6.0-fold in FGF-2+EGF. The reduced population expansion persisted with subsequent passages resulting in a gradual decline in total cells and failure to generate neurospheres beyond passage 6.

To confirm that neurospheres generated under these conditions formed through clonal expansion of single cells rather than via re-aggregation of dissociated cells, we performed serial dilution clonal analysis using FGF-2+EGF-generated neurospheres. At each passage, cells were cultured by serial dilution ranging from 1000 to 62.5 cells/cm^2^ and the number of neurospheres/well was counted after 15 DIV. Although clonogenicity was not influenced by seeding density, demonstrating the clonal origin of neurospheres (data not shown), clonogenicity decreased markedly with each passage, decreasing 10-fold from passage 1 to 2 (**Fig. 4B**) which accounts for the reduced cell yields observed in **Fig. 4A**. Concomitant with reduced clonogenicity over successive passages, assessment of neurosphere multipotency revealed that the capacity to generate MAP2^+^ neurons (**Fig. 4C**) and O4^+^ oligodendrocytes (**Fig. 4D**) was retained for the first 3 passages, beyond which only GFAP^+^ astrocytes were identified (**Fig. 4E**).

## Discussion

We describe for the first time an *in vitro* method to identify multipotent FGF-2- and EGF-responsive NPCs residing within V1 neocortex of the postnatal nonhuman primate. Neurospheres expressed neural progenitor markers Sox2 and nestin, were mitotically active as demonstrated by Ki67 expression and BrdU incorporation, and exhibited limited self-renewal for up to 6 passages. Under differentiating conditions, neurospheres generated neurons, oligodendrocytes and astrocytes up to passage 3 after which only astrocytes were identified. Declining clonogenicity with successive passaging was indicative of reduced self-renewing cell divisions that resulted in an ever-decreasing rate of population expansion. Consequently, most cultures could not be maintained beyond passage 6. Collectively our data suggest that a population of NPCs are retained within the postnatal V1 which exhibit limited self-renewal and multipotential differentiation *in vitro*. The decay observed after an initial phase of intense proliferation could be the consequence of asymmetrical or terminal symmetrical divisions of the NPCs that give rise to one or two quiescent progenitors respectively, depleting the population at each cycle.

### Origin of V1 neurosphere-forming cells

Immunohistochemical analyses identified heterogenous cell populations within V1 that could potentially represent the *in vivo* neurosphere-forming cell population. NG2^+^ progenitors and GFAP^+^ astrocytes expressing the Sox2 transcription factor were distributed homogeneously throughout all cortical layers and white matter. Sox2 expression is sustained in neurogenic regions of the embryonic and adult CNS [28] and is sufficient to maintain cells in a neural progenitor state [29]. A subpopulation of Sox2^+^ cells in V1 co-expressed the proliferation marker Ki67 and subsets of both GFAP^+^ cells and NG2^+^ glia expressed Ki67 suggesting that these cell populations are proliferative *in vivo* and could plausibly be responsive to EGF and/or FGF-2 *in vitro*. This hypothesis is supported by previous reports demonstrating that NG2 also labels a population of multipotent NPCs in the postnatal CNS [30], and NSCs in the adult SVZ express GFAP [31] and GFAP^+^ cells isolated from the injured neocortex generate multipotent neurospheres *in vitro* [16].

We have identified two anatomically distinct populations of DCX^+^ cells. DCX is expressed by rapidly dividing neuroblasts located in adult neurogenic compartments and is downregulated upon neuronal maturation [32]. A population of DCX^+^ cells that divides into quiescent precursors has recently been reported in the non-human primate [33]. By contrast we did not identify any DCX^+^ cells that co-expressed Ki67 suggesting that DCX^+^ cells V1 are predominantly quiescent *in vivo* although we cannot exclude the possibility that they re-enter the cell cycle *in vitro*. The absence of cells expressing Tbr2^+^, a marker of intermediate neuronal progenitors in the rodent and marmoset SVZ [34], [35], is consistent with the view that SVZ-derived intermediate neuronal progenitors are not present in V1 at PD14. It should be noted that we have not qualified all subpopulations of SVZ progenitors in PD14 marmoset tissue. For instance, in addition to NG2, transit amplifying progenitors (Type C cells) that give rise to neuroblasts (type A cells) in the postnatal rodent SVZ also express Dlx2 and Mash1/Ascl1, markers that are commonly used to identify these progenitor cells *in vivo* [36]–[38]. Although we have not directly assessed expression of Dlx2 and Mash1/Ascl1 in postnatal marmoset V1, excised V1 tissue is not expected to contain any contaminating SVZ tissue. The most caudal aspect of the lateral ventricles defined by histological analysis [24] and T2-weighted magnetic resonance imaging (MRI) [39], is at least 6.5 mm anterior to the point of V1 excision. Thus even though the entire rostrocaudal extent of the mouse SVZ is capable of generating neurons *in vivo* [40], excised V1 is several millimetres away from the most caudal aspect of this germinal zone.

Definitive experiments to unambiguously identify the neurosphere-forming cell population in V1 will require the development of approaches to selectively label all putative neurosphere-forming populations, including but not limited to Sox2^+^, Ng2^+^, GFAP^+^ and DCX^+^. Notably, a number of distinct cell populations have been demonstrated to exhibit the capacity to generate multipotent neurospheres *in vitro*. For instance, adult rat spinal cord meningeal cells can generate neurospheres when cultured in the presence of FGF-2 but produce few astrocytes upon mitogen withdrawal [41]. V1-derived neurosphere-froming cells are unlikely to derive from the meninges since this structure was carefully removed from excised marmoset V1 tissue prior to culture and the neurospheres derived exhibited robust astroglial differentiation. Another study reported the generation of multipotent neurospheres from NG2-positive pericytes isolated from the adult rat brain [42], a specialised mesenchymal cell, and putative source of mesenchymal stem cells [43]. Although pericyte-derived neurospheres express neuronal, oligodendroglial and astroglial markers after differentiation, most cells co-express antigens for at least two of these three distinct lineages and exhibit cell morphologies indicative of intermediate differentiation [42]. At present such an approach is challenging due to the limited availability of suitable cell surface markers or reporters of promoter activity in the marmoset neocortex.

### Characteristics of V1 NPCs and role of the growth factors

Our results reveal distinct roles for EGF and FGF-2 upon V1 NPCs *in vitro*. Both factors promoted the formation of neurospheres, however EGF-generated neurospheres were consistently smaller and generated significantly fewer oligodendrocytes. These results suggest that both EGF and FGF-2 are mitogens for neurosphere-forming cells, but FGF-2 potentiates NPC proliferation and the generation of oligodendrocyte progenitor cells. Consistent with previous studies in the rodent [44], the V1-derived NPCs were principally generated astrocytes. Despite the increased yield of FGF-2/EGF- over FGF-2-generated neurosphere cells, the total projected number of neurons that would be generated were the entire population of primary neurosphere cells subjected to *in vitro* differentiation remains higher for FGF-2-generated neurosphere cells. Thus we speculate that the combinatorial use of FGF-2 and EGF promotes increased survival of NPCs over FGF-2 alone but that FGF-2 remains the primary driver of differentiated cell fate *in vitro*.

### In vivo function of FGF-2/EGF-responsive neurosphere-forming cells

Several studies suggest that classically non-neurogenic regions of the brain including the neocortex exhibit latent neurogenic potential that is repressed under normal physiological conditions [15], [16]. While the evidence for postnatal neurogenesis in the nonpathological neocortex remains contested [1], [3], [11], a body of evidence is emerging that neurogenesis can occur after injury in the nonhuman primate [45]. The EGF/FGF-2-responsive NPCs that we have identified in V1 define a population of resident multipotent neocortical NPCs that could plausibly participate in low-level neurogenesis under normal physiological conditions and/or participate in local regenerative responses following injury. Alternatively, neurogenesis could occur to a greater extent than initially appreciated but the neurons could fail to survive long term [46], [47]. These data have two important implications: first, NPC migration from the SVZ is not essential for neocortical neurogenesis, an issue that becomes increasingly significant in the human where distances between the classical neurogenic niches and the neocortex are far greater; second, strategies that activate resident neocortical NPCs *in situ* could be employed to potentiate an endogenous response to injury.

### Marmoset monkey as a model organism for studying postnatal neurogenesis

We demonstrate the increasing utility of the marmoset monkey as a powerful nonhuman primate model for the assessment of postnatal neurogenesis and its suitability for both *in vivo* and *in vitro* analysis. The SVZ of the marmoset has recently been demonstrated to exhibit close similarity to the human SVZ [39]. Moreover, this species offers several advantages over other nonhuman primates including their small size (<500 g), a lissencephalic neocortex which undergoes isotropic growth and their utility for functional and anatomical studies of brain development, activity and repair [21], [22], [25], [48]. We propose that specific studies employing the marmoset model following neocortical injury will provide an excellent model for translational research strategies targeting endogenous NPCs to promote neural repair in man.

## References

[1] Rakic P (2002). Neurogenesis in adult primate neocortex: an evaluation of the evidence.. Nat Rev Neurosci.

[2] Ming G-L, Song H (2011). Adult neurogenesis in the Mammalian brain: significant answers and significant questions.. Neuron.

[3] Gould E (2007). How widespread is adult neurogenesis in mammals?. Nat Rev Neurosci.

[4] Bernier PJ, Bédard A, Vinet J, Levesque M, Parent A (2002). Newly generated neurons in the amygdala and adjoining cortex of adult primates.. Proc Natl Acad Sci USA.

[5] Dayer AG, Cleaver KM, Abouantoun T, Cameron HA (2005). New GABAergic interneurons in the adult neocortex and striatum are generated from different precursors.. J Cell Biol.

[6] Gould E, Reeves AJ, Graziano MS, Gross CG (1999). Neurogenesis in the neocortex of adult primates.. Science.

[7] Kaplan MS (1981). Neurogenesis in the 3-month-old rat visual cortex.. J Comp Neurol.

[8] Bhardwaj RD, Curtis MA, Spalding KL, Buchholz BA, Fink D (2006). Neocortical neurogenesis in humans is restricted to development.. Proc Natl Acad Sci USA.

[9] Koketsu D, Mikami A, Miyamoto Y, Hisatsune T (2003). Nonrenewal of neurons in the cerebral neocortex of adult macaque monkeys.. Journal of Neuroscience.

[10] Kornack DR, Rakic P (2001). Cell proliferation without neurogenesis in adult primate neocortex.. Science.

[11] Cameron HA, Dayer AG (2008). New interneurons in the adult neocortex: small, sparse, but significant?. Biol Psychiatry.

[12] Gritti A, Parati EA, Cova L, Frolichsthal P, Galli R (1996). Multipotential stem cells from the adult mouse brain proliferate and self-renew in response to basic fibroblast growth factor.. J Neurosci.

[13] Reynolds BA, Weiss S (1992). Generation of neurons and astrocytes from isolated cells of the adult mammalian central nervous system.. Science.

[14] Walker TL, White A, Black DM, Wallace RH, Sah P (2008). Latent stem and progenitor cells in the hippocampus are activated by neural excitation.. Journal of Neuroscience.

[15] Palmer TD, Markakis EA, Willhoite AR, Safar F, Gage FH (1999). Fibroblast growth factor-2 activates a latent neurogenic program in neural stem cells from diverse regions of the adult CNS.. Journal of Neuroscience.

[16] Buffo A, Rite I, Tripathi P, Lepier A, Colak D (2008). Origin and progeny of reactive gliosis: A source of multipotent cells in the injured brain.. Proc Natl Acad Sci USA.

[17] Smart IHM, Dehay C, Giroud P, Berland M, Kennedy H (2002). Unique morphological features of the proliferative zones and postmitotic compartments of the neural epithelium giving rise to striate and extrastriate cortex in the monkey.. Cereb Cortex.

[18] Missler M, Wolff A, Merker HJ, Wolff JR (1993). Pre- and postnatal development of the primary visual cortex of the common marmoset. II. Formation, remodelling, and elimination of synapses as overlapping processes.. J Comp Neurol.

[19] Bourne JA, Rosa MGP (2006). Hierarchical development of the primate visual cortex, as revealed by neurofilament immunoreactivity: early maturation of the middle temporal area (MT).. Cereb Cortex.

[20] Rakic P (2002). Pre-and post-developmental neurogenesis in primates.. Clinical Neuroscience Research.

[21] Fritschy JM, Garey LJ (1986). Quantitative changes in morphological parameters in the developing visual cortex of the marmoset monkey.. Brain Res.

[22] Bourne JA, Warner CE, Rosa MGP (2005). Topographic and laminar maturation of striate cortex in early postnatal marmoset monkeys, as revealed by neurofilament immunohistochemistry.. Cereb Cortex.

[23] Sirko S, Neitz A, Mittmann T, Horvat-Bröcker A, Holst von A (2009). Focal laser-lesions activate an endogenous population of neural stem/progenitor cells in the adult visual cortex.. Brain.

[24] Palazzi X, Bordier N (2008). The Marmoset Brain in Stereotaxic Coordinates.. The Marmoset Brain in Stereotaxic Coordinates.

[25] Goldshmit Y, Bourne J (2010). Upregulation of EphA4 on astrocytes potentially mediates astrocytic gliosis after cortical lesion in the marmoset monkey.. J Neurotrauma.

[26] Merson TD, Dixon MP, Collin C, Rietze RL, Bartlett PF (2006). The transcriptional coactivator Querkopf controls adult neurogenesis.. Journal of Neuroscience.

[27] Bylund M, Andersson E, Novitch BG, Muhr J (2003). Vertebrate neurogenesis is counteracted by Sox1-3 activity.. Nat Neurosci.

[28] Brazel CY, Limke TL, Osborne JK, Miura T, Cai J (2005). Sox2 expression defines a heterogeneous population of neurosphere-forming cells in the adult murine brain.. Aging Cell.

[29] Bylund M, Andersson E, Novitch BG, Muhr J (2003). Vertebrate neurogenesis is counteracted by Sox1-3 activity.. Nat Neurosci.

[30] Belachew S, Chittajallu R, Aguirre AA, Yuan X, Kirby M (2003). Postnatal NG2 proteoglycan-expressing progenitor cells are intrinsically multipotent and generate functional neurons.. J Cell Biol.

[31] Doetsch F, Caillé I, Lim DA, García-Verdugo JM, Alvarez-Buylla A (1999). Subventricular zone astrocytes are neural stem cells in the adult mammalian brain.. Cell.

[32] Brown JP, Couillard-Despres S, Cooper-Kuhn CM, Winkler J, Aigner L (2003). Transient expression of doublecortin during adult neurogenesis.. J Comp Neurol.

[33] Bloch J, Kaeser M, Sadeghi Y, Rouiller EM, Redmond DE (2011). Doublecortin-positive cells in the adult primate cerebral cortex and possible role in brain plasticity and development.. J Comp Neurol.

[34] Kowalczyk T, Pontious A, Englund C, Daza RAM, Bedogni F (2009). Intermediate neuronal progenitors (basal progenitors) produce pyramidal-projection neurons for all layers of cerebral cortex.. Cerebral Cortex.

[35] García-Moreno F, Vasistha NA, Trevia N, Bourne JA, Molnár Z (2012). Compartmentalization of cerebral cortical germinal zones in a lissencephalic primate and gyrencephalic rodent.. Cerebral Cortex.

[36] Doetsch F, Petreanu L, Caille I, Garcia-Verdugo JM, Alvarez-Buylla A (2002). EGF converts transit-amplifying neurogenic precursors in the adult brain into multipotent stem cells.. Neuron.

[37] Aguirre AA, Chittajallu R, Belachew S, Gallo V (2004). NG2-expressing cells in the subventricular zone are type C-like cells and contribute to interneuron generation in the postnatal hippocampus.. J Cell Biol.

[38] Parras CM, Galli R, Britz O, Soares S, Galichet C (2004). Mash1 specifies neurons and oligodendrocytes in the postnatal brain.. EMBO J.

[39] Sawamoto K, Hirota Y, Alfaro-Cervello C, Soriano-Navarro M, He X (2011). Cellular composition and organization of the subventricular zone and rostral migratory stream in the adult and neonatal common marmoset brain.. J Comp Neurol.

[40] Merkle FT, Mirzadeh Z, Alvarez-Buylla A (2007). Mosaic organization of neural stem cells in the adult brain.. Science.

[41] Decimo I, Bifari F, Francisco JR, Malpeli G, Dolci S (2011). Nestin- and DCX-Positive Cells Reside in Adult Spinal Cord Meninges and Participate to Injury-Induced Parenchymal Reaction.. STEM CELLS.

[42] Dore-Duffy P, Katychev A, Wang X, Van Buren E (2006). CNS microvascular pericytes exhibit multipotential stem cell activity.. Journal of Cerebral Blood Flow & Metabolism.

[43] Crisan M, Yap S, Casteilla L, Chen C-W, Corselli M (2008). A perivascular origin for mesenchymal stem cells in multiple human organs.. Cell Stem Cell.

[44] Qian X, Shen Q, Goderie SK, He W, Capela A (2000). Timing of CNS cell generation: a programmed sequence of neuron and glial cell production from isolated murine cortical stem cells.. Neuron.

[45] Vessal M, Darian-Smith C (2010). Adult neurogenesis occurs in primate sensorimotor cortex following cervical dorsal rhizotomy.. Journal of Neuroscience.

[46] Takemura NU (2005). Evidence for neurogenesis within the white matter beneath the temporal neocortex of the adult rat brain.. NSC.

[47] Kim WR, Chun SK, Kim TW, Kim H, Ono K (2011). Evidence for the spontaneous production but massive programmed cell death of new neurons in the subcallosal zone of the postnatal mouse brain.. Eur J Neurosci.

[48] Garey LJ, de Courten C (1983). Structural development of the lateral geniculate nucleus and visual cortex in monkey and man.. Behav Brain Res.

